# Impact of COVID-19 epidemic on antihypertensive drug treatment disruptions: results from a nationwide interrupted time-series analysis

**DOI:** 10.3389/fphar.2023.1129244

**Published:** 2023-05-15

**Authors:** Clément Mathieu, Julien Bezin, Antoine Pariente

**Affiliations:** ^1^ Inserm, Bordeaux Population Health Research Center, Team AHeaD, UMR 1219, University Bordeaux, Bordeaux, France; ^2^ CHU de Bordeaux, Service de Pharmacologie Médicale, Bordeaux, France

**Keywords:** COVID-19, pharmacoepidemiology, impact assessment, antihypertensive drug, cardiovascular drugs, treatment disruption, interrupted time series

## Abstract

**Background:** The COVID-19 epidemic has disrupted care and access to care in many ways. It was accompanied by an excess of cardiovascular drug treatment discontinuations. We sought to investigate a deeper potential impact of the COVID-19 epidemic on antihypertensive drug treatment disruptions by assessing whether the epidemic induced some changes in the characteristics of disruptions in terms of duration, treatment outcome, and patient characteristics.

**Methods:** From March 2018 to February 2021, a repeated cohort analysis was performed using French national health insurance databases. The impact of the epidemic on treatment discontinuations and resumption of antihypertensive medications was assessed using preformed interrupted time series analyses either on a quarterly basis.

**Results:** Among all adult patients on antihypertensive medication, we identified 2,318,844 (18.7%) who discontinued their antihypertensive treatment during the first blocking period in France. No differences were observed between periods in the characteristics of patients who interrupted their treatment or in the duration of treatment disruptions. The COVID-19 epidemic was not accompanied by a change in the proportion of patients who fully resumed treatment after a disruption, neither in level nor in trend/slope [change in level: 2.66 (−0.11; 5.42); change in slope: −0.67 (−1.54; 0.20)]. Results were similar for the proportion of patients who permanently discontinued treatment within 1 year of disruption [level change: −0.21 (−2.08; 1.65); slope change: 0.24 (−0.40; 0.87)].

**Conclusion:** This study showed that, although it led to an increase in cardiovascular drug disruptions, the COVID-19 epidemic did not change the characteristics of these. First, disruptions were not prolonged, and post-disruption treatment outcomes remained unchanged. Second, patients who experienced antihypertensive drug disruptions during the COVID-19 outbreak were essentially similar to those who experienced disruptions before it.

## Introduction

Severe acute respiratory syndrome coronavirus 2 (SARS-CoV-2) infection first emerged in China in late 2019 and quickly evolved into a global pandemic, with the virus rapidly spreading throughout France, infecting 34 million people and killing more than 160,000 by 1 September 2022. In addition to its direct health impact, the COVID-19 pandemic proved to be a critical threat to healthcare systems where saturation of emergency care led to an unprecedented health crisis, whether in terms of deaths (Islam et al., 2021; Salinas et al., 2021), hospitalizations (De Filippo et al., 2020; Mesnier et al., 2020; Bogh et al., 2021), or disruption of care (Mejía et al., 2020; Cransac-Miet et al., 2021). The impact of the pandemic was obviously not limited to hospitals (Moreno et al., 2020; Salari et al., 2020); the disorganization of access to care linked to the pandemic, either directly or through the mitigating measures adopted, also affected the out-of-hospital setting, including movement restrictions or lockdowns (Cauchemez et al., 2020; Spaccaferri et al., 2020). The massive flow of Sars-CoV-2 infected patients requiring emergency or even intensive care has directly competed with the management of other patients (Kadri et al., 2021; Dichter et al., 2022), such as those with chronic cardiovascular disease (De Filippo et al., 2020; De Rosa et al., 2020; Marijon et al., 2020; Mesnier et al., 2020). In order to optimize the use of the healthcare system, to try to save as much as possible some healthcare resources for non-COVID patients, and to preserve access to care, many tools were put in place during the epidemic, including deprogramming periods for elective hospital admissions (Baudry et al., 2021), increased access to telemedicine (Kamel et al., 2021; Nan et al., 2021), or the extension of the maximum duration of prescriptions, for example, (Ministère des solidarités et de la santé, 2020). While they are certainly effective, they could not prevent all the consequences of such a massive phenomenon as the epidemic and its mitigating measures on maintaining the usual level of access to care for patients with chronic diseases during the COVID period. In a previous study, we investigated the impact of the COVID-19 epidemic and its containment measures on the use of cardiovascular medications, and were able to demonstrate that the period was accompanied by both an excess of treatment disruptions and a significant decrease in treatment initiations, especially during the first lock-in, particularly for antihypertensive medications (Mathieu et al., 2022). To investigate this impact further, we next sought to study whether the treatment disruptions observed during the COVID-19 epidemic in France differed from those usually observed and associated with compromised efficacy. If true, this could indicate that the treatment disruptions observed during the epidemic may have resulted in a different risk of cardiovascular complications, independent of their increased frequency.

## Materials and methods

### Data source

Individualized medico-administrative data were extracted from the National Health Data System (SNDS, formerly SNIIRAM) (Tuppin et al., 2010; Bezin et al., 2017), coupled with the national database of hospital discharges (PMSI), which covers the entire French population, i.e., 67 million inhabitants, and which has been widely used in France to conduct pharmaco-epidemiological studies, notably on the COVID-19 epidemic (Semenzato et al., 2021; Le Bihan Benjamin et al., 2022; Semenzato et al., 2022). The database consists of the anonymous and exhaustive recording of all reimbursements for outpatient health expenses, laboratory tests and prescribed drugs. The health expenses of people with a long-term condition (LTDs), such as cancer or diabetes, are fully covered and their diagnoses are recorded according to the International Classification of Diseases, 10th revision (ICD-10). Medical diagnoses are coded according to the ICD-10 classification and the main medical or surgical procedures are coded according to the *Classification Commune des Actes Medicaux* (CCAM) (CCAM, 2022; ICD, 2022).

### Study design and population

We conducted a weekly repeated cohort study at the national level using data from the SNDS. The present study focused on beneficiaries of the general health insurance scheme, which covers 88% of the French population. This scheme, whose official name can be confusing, is the affiliation scheme for all salaried workers, unemployed, students or retirees not affiliated to one of the existing specific schemes (mainly dedicated to farmers, dockers, clergymen).

The study period of interest included all weeks in the period from January 2018 to December 2020. For each of the study weeks, patients who received at least one of the antihypertensive drug deliveries during the study period were eligible if they had been affiliated with the French general health insurance scheme and present in the database at least 365 days before the start of the week (presence evidenced by the identification of at least one reimbursement for any care 365 days or more before the start of the week) and were alive and aged 18 years or older on the first day of the week. This led to the definition of as many subcohorts/cohorts of interest as weeks studied (week subcohorts; total: *n* = 156), as described previously (Mathieu et al., 2022).

### Drugs of interest and exposure assessment

The five drug classes studied were beta-blockers, angiotensin-converting enzyme (ACE) inhibitors, angiotensin II receptor blockers (ARBs), calcium channel blockers (CCBs), and thiazide diuretics. Information on out-of-hospital dispensing of all drugs in these classes was identified in the SNDS database using the corresponding anatomic therapeutic chemical (ATC) classification codes over the entire period from week 26 of 2017 to week 8 of 2022 (Supplementary Material S1).

**FIGURE 1:**
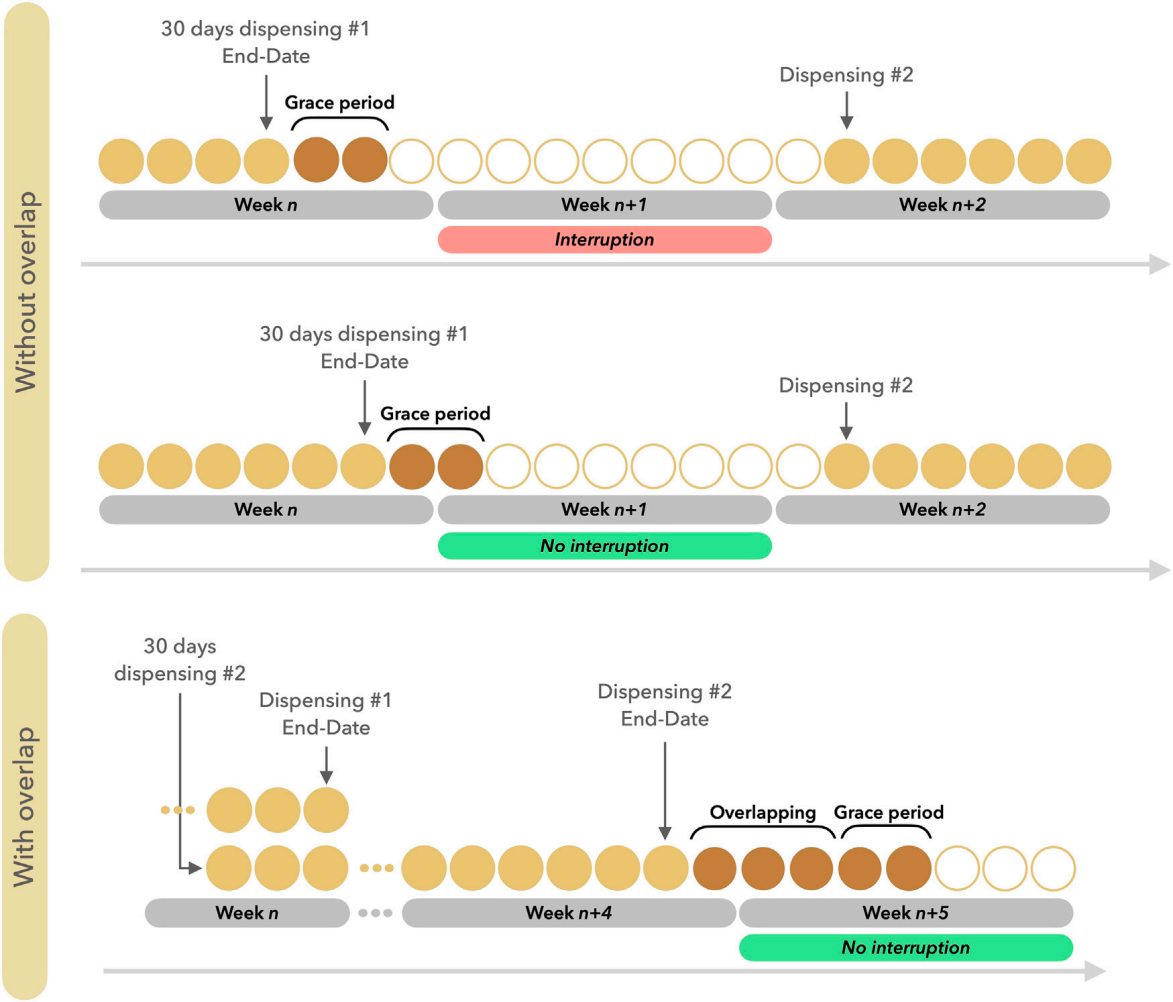
Illustration defining the concepts used to define drug treatment disruptions.

For each patient in a given week’s subcohort, exposure to each individual drug in these classes was estimated using dispensing information from the week of interest and the previous 26 weeks (i.e., previous 6 months to allow estimation of potential storage). For each dispensing, the covered treatment period was considered to start on the date of dispensing and last for 30 days (or 91 days for quarterly packs), plus a 5% grace period as is usually done (Pazzagli et al., 2022). When the periods covered by two or more dispensations overlapped, the number of overlapping days was added to the length of the period covered by the last dispensation to account for possible stockpiling. The occurrence of a period not covered by the treatment, after the end of the period covered by the last dispensation (plus the grace period and potential storage), constituted a treatment disruption. In other words, a minimum period of 7 days without treatment constituted a disruption. We proposed instead illustrating how disruptions were considered using the following figure (Figure 1).

### Outcome measures

**FIGURE 2:**
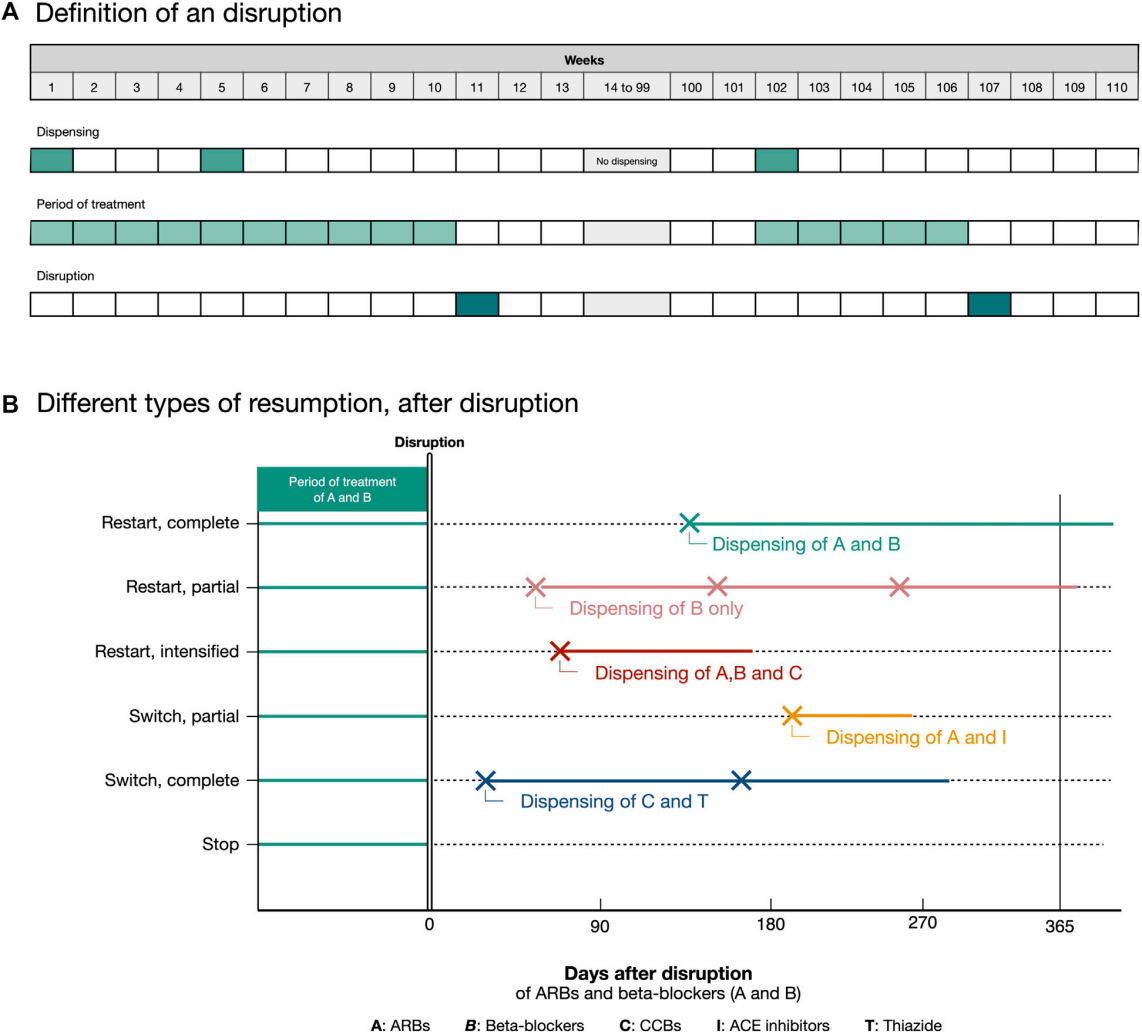
Definitions of an antihypertensive drugs disruption **(A)**, and definition of the different outcomes for treatment after disruption **(B)**.

Because the incidence of treatment disruptions was studied in a previous large-scale analysis of the impact of the COVID-19 epidemic on cardiovascular drug utilization (Mathieu et al., 2022), here we investigated the characteristics of weekly treatment disruptions in terms of duration (in days) and outcome. Based on the assessment of treatment utilization in the calendar year following an antihypertensive drug disruption, this outcome could be classified as follows (Figure 2):

- Restart, complete: all interrupted drug classes resumed;
- Restart, intensified: all discontinued drug classes were restarted and one or more additional drug classes were initiated;
- Restart, partial: only part of the interrupted drug classes were restarted and no new drug classes were initiated;
- Switch, partial: some of the discontinued drug classes were restarted and one or more other drug classes were initiated;
- Switch, complete: no interrupted drug classes were resumed and one or more additional drug classes were initiated;
- Stop: no interrupted drug classes were restarted and no new drug classes were initiated.

### Statistical analysis

The study of the impact of the COVID-19 epidemic on the characteristics of antihypertensive drug therapy disruptions examined both the characteristics of the identified disruptions and those of the patients experiencing disruptions. Because a patient could contribute to multiple quarterly subcohorts, the characteristics of eligible patients were described on an annual basis beginning with the March-May 2018, 2019, and 2020 quarter. This description took into account age, gender, the existence of a severe long-term condition, socioeconomic vulnerability assessed using the French deprivation index (FDep), a composite indicator that characterizes the socioeconomic status of families to account for territorial disparities (Rey et al., 2009; Barry et al., 2022). Because drug use was not measurable during hospital stays and its rate potentially varied over the period (Suissa, 2008; Bezin et al., 2017), the occurrence of hospitalizations was assessed over the 1-month and 3-month period preceding antihypertensive drug breaks.

Finally, mortality rates within 1 month or 1 year of treatment disruptions were estimated.

To investigate the impact of the COVID-19 epidemic on treatment outcomes after discontinuation, we performed interrupted time series (ITS) regressions (Penfold and Zhang, 2013; Zhang et al., 2020; Jiang et al., 2022). ITS explores the impact of an event (e.g., in our case, the COVID-19 epidemic) on time series data, i.e., repeated measurements of a given outcome at regular intervals (e.g., monthly or quarterly) (Pariente et al., 2017). The impact is estimated in terms of potential level change and trend. The level change represents the immediate and permanent change caused by the epidemic; the slope change represents the time-dependent effect due to the epidemic. In the analysis we performed, the pre-epidemic period considered data from 5 March 2018, to 1 March 2020, and the epidemic period data from 2 March 2020 (the fixed breakpoint for the analysis), to 6 December 2020. Epidemic effect estimates were expressed in terms of rate ratios for the different treatment outcomes studied and provided with their 95% confidence intervals (95% CI).

Additional analyses were performed to account for gender, to assess the epidemic impact of COVID-19 on the post-breakthrough treatment outcome assessed at 6 months instead of 1 year, to assess the epidemic impact of COVID-19 on the treatment outcome according to each pharmacological drug class of interest, and to assess the epidemic impact of COVID-19 on a quarterly basis instead of a weekly basis. Data management and analyses were performed using SAS version 9.4 software (SAS Institute, United States).

## Results

More than 12 million patients registered in the French SNDS national databases met the study inclusion criteria in the first quarter of 2020. Over the study period, the population size remained stable across all quarters studied. Of these, 2,318 844 patients on antihypertensive medication discontinued treatment (18.7%), 55.5% of whom were women (Table 1); the median age was 71.3 years (Inter-Quartile Range, IQR: 61.5–80.8). Of all patients who discontinued treatment during March-May 2020, 60.9% resumed exactly the same treatment (restart, complete) after a mean disruption of 67.6 days. The distribution of the French deprivation index quintiles did not vary over the study period.

**TABLE 1:** Characteristics of patients who presented an antihypertensive drug a disruption during the 2018, 2019 and 2020 March to May quarters.

	March-May period		
	2018	2019	2020
	*N* = 2,110,354	*N* = 2,187,878	*N* = 2,318,844
**Age**—year, *median [IQR]*	70.2 (60.7–80.0)	71.1 (61.3–80.9)	71.3 (61.5–80.8)
**Sex**—female, *No. (%)*	1,169,001 (55.4)	1,212,365 (55.4)	1,285,865 (55.5)
**Hospitalisation before disruption**, *No. (%)*			
Within 1 month	98,926 (4.7)	114,265 (5.2)	87,237 (3.8)
Within 3 months	206,334 (9.8)	230,195 (10.5)	195,029 (8.4)
**French Deprivation Index**, *No. (%)*			
1st quintile	356,872 (16.9)	368,520 (16.8)	390,369 (16.8)
2nd quintile	370,489 (17.6)	381,784 (17.4)	411,212 (17.7)
3rd quintile	408,127 (19.3)	419,738 (19.2)	450,608 (19.4)
4th quintile	433,199 (20.5)	446,567 (20.4)	476,913 (20.6)
5th quintile	460,332 (21.8)	474,034 (21.7)	500,525 (21.6)
**Long-term disease**, *No. (%)*	1,249,032 (59.2)	1,317,531 (60.2)	1,386,436 (59.8)
Disabling stroke	72,027 (3.4)	79,809 (3.6)	84,145 (3.6)
Bone marrow failure and other chronic cytopenias	2,997 (0.1)	3,515 (0.2)	3,568 (0.2)
Chronic arterial diseases with ischemic events	89,070 (4.2)	95,796 (4.4)	98,522 (4.2)
Complicated schistosomiasis	19 (0.0)	18 (0.0)	22 (0.0)
Severe heart failure, severe rhythm disorders, severe valvular heart disease, severe congenital heart disease	220,366 (10.4)	244,274 (11.2)	253,308 (10.9)
Chronic active liver diseases and cirrhosis	16,682 (0.8)	18,203 (0.8)	18,429 (0.8)
Severe primary immune deficiency requiring prolonged treatment, HIV infection	5,359 (0.3)	5,996 (0.3)	6,638 (0.3)
Types 1 and 2 diabetes	410,082 (19.4)	444,986 (20.3)	472,157 (20.4)
Severe forms of neurological and muscular diseases (including myopathy), severe epilepsy	19,550 (0.9)	21,937 (1.0)	23,215 (1.0)
Hemoglobinopathy, severe constitutional and acquired chronic hemolysis	715 (0.0)	786 (0.0)	820 (0.0)
Hemophilias and constitutional disorders of severe hemostasis	3,130 (0.1)	3,404 (0.2)	3,720 (0.2)
Severe arterial hypertension	105,549 (5.0)	103,674 (4.7)	96,269 (4.2)
Coronary disease	241,337 (11.4)	256,429 (11.7)	264,446 (11.4)
Severe chronic respiratory failure	40,289 (1.9)	42,591 (1.9)	40,747 (1.8)
Alzheimer’s disease and other dementias	42,210 (2.0)	49,512 (2.3)	48,909 (2.1)
Parkison’s disease	14,691 (0.7)	16,600 (0.8)	17,061 (0.7)
Inherited metabolic diseases requiring prolonged specialized treatment	6,265 (0.3)	6,455 (0.3)	6,755 (0.3)
Cystic fibrosis	165 (0.0)	184 (0.0)	188 (0.0)
Severe chronic kidney disease and primary nephrotic syndrome	37,436 (1.8)	41,339 (1.9)	43,562 (1.9)
Paraplegia	1,839 (0.1)	1,960 (0.1)	2,110 (0.1)
Vasculitis, systemic lupus erythematosus, systemic scleroderma	13,783 (0.7)	14,606 (0.7)	15,763 (0.7)
Progressive rheumatoid arthritis	22,345 (1.1)	24,085 (1.1)	25,204 (1.1)
Long-term psychiatric disorders	81,722 (3.9)	88,279 (4.0)	88,643 (3.8)
Ulcerative colitis and Crohn’s disease	7,441 (0.4)	8,003 (0.4)	8,833 (0.4)
Multiple sclerosis	3,689 (0.2)	4,098 (0.2)	4,450 (0.2)
Structural idiopathic scoliosis evolving to spinal maturation	1,449 (0.1)	1,606 (0.1)	1,776 (0.1)
Serious spondylitis	10,177 (0.5)	11,242 (0.5)	11,967 (0.5)
Following transplants	2,445 (0.1)	2,669 (0.1)	2,868 (0.1)
Active tuberculosis, leprosy	719 (0.0)	762 (0.0)	802 (0.0)
Malignant neoplasm, malignant disease of the lymphatic or hematopoietic tissue	220,865 (10.5)	247,741 (11.3)	256,319 (11.1)

**FIGURE 3:**
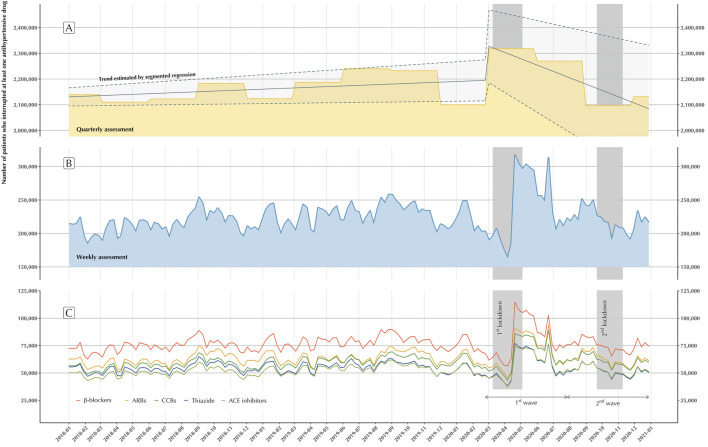
Time series of the number of patients who had at least one disruption of antihypertensive drugs, per quarter **(A)**, per week **(B)**, and per according to antihypertensive drug categories **(C)**, between March 2018 and December 2021, in France.

Our previously published results were replicated (Mathieu et al., 2022). Prior to the COVID-19 era, time series showed a limited and consistent increase in treatment disruptions over time, regardless of pharmacological class, whether the analysis considered quarterly or weekly assessments (Figures 3A, B). Beta-blockers were the most frequently discontinued antihypertensive drugs, with approximately 75,000 weekly disruptions through February 2020, with ACE inhibitors being the second most discontinued class (with approximately 50,000 weekly disruptions through February 2020; Figure 3C). The onset of the COVID-19 epidemic, including the first lock-in period, was accompanied by changes in the weekly incidence of antihypertensive treatment disruptions, with an increase in their frequency in the last weeks of the lock-in period and those that followed (Figure 3B) that affected all pharmacological classes (Figure 3C). This increase markedly affected the first two quarters of the epidemic in France (Figure 3A).

No differences in characteristics were found between subjects who discontinued antihypertensive treatment in the quarter after the start of the COVID-19 epidemic in 2020 in France and in the corresponding quarters of 2018 and 2019 (proportion of women: 55.4% in 2018 and 2019; median age: 70.2 years (IQR: 60.7–80.0) in 2018 and 71.1 years (IQR: 61.3–80.9) in 2019) (Table 1). The most frequent comorbidities recorded as serious long-term conditions among the considered affiliates were also similarly distributed among subjects discontinuing treatment in the same 2020, 2019, and 2018 periods (e.g., type 1 or type 2 diabetes mellitus: 20.4%, 20.3%, 19.4%; malignant neoplasm and diseases of the lymphatic or hematopoietic system: 11.1%, 11.3%, 10.5%; coronary heart disease: 11.4%, 11.7%, 11.4%). The distribution of the FDep index also appeared comparable between all years (Table 1). Among subjects who discontinued treatment in the quarter after the start of the COVID-19 epidemic in France in 2020, 3.8% had been hospitalized in the previous month and 8.4% in the previous 3 months; these rates were lower than in previous years. The incidence of death within 1 month or 1 year of a disruption of antihypertensive treatment was 53 and 565 per 10,000 individuals, potentially higher than that observed after disruptions in the corresponding quarters of 2018 and 2019 (49 and 543 per 10,000; 47 and 519 per 10,000).

**FIGURE 4:**
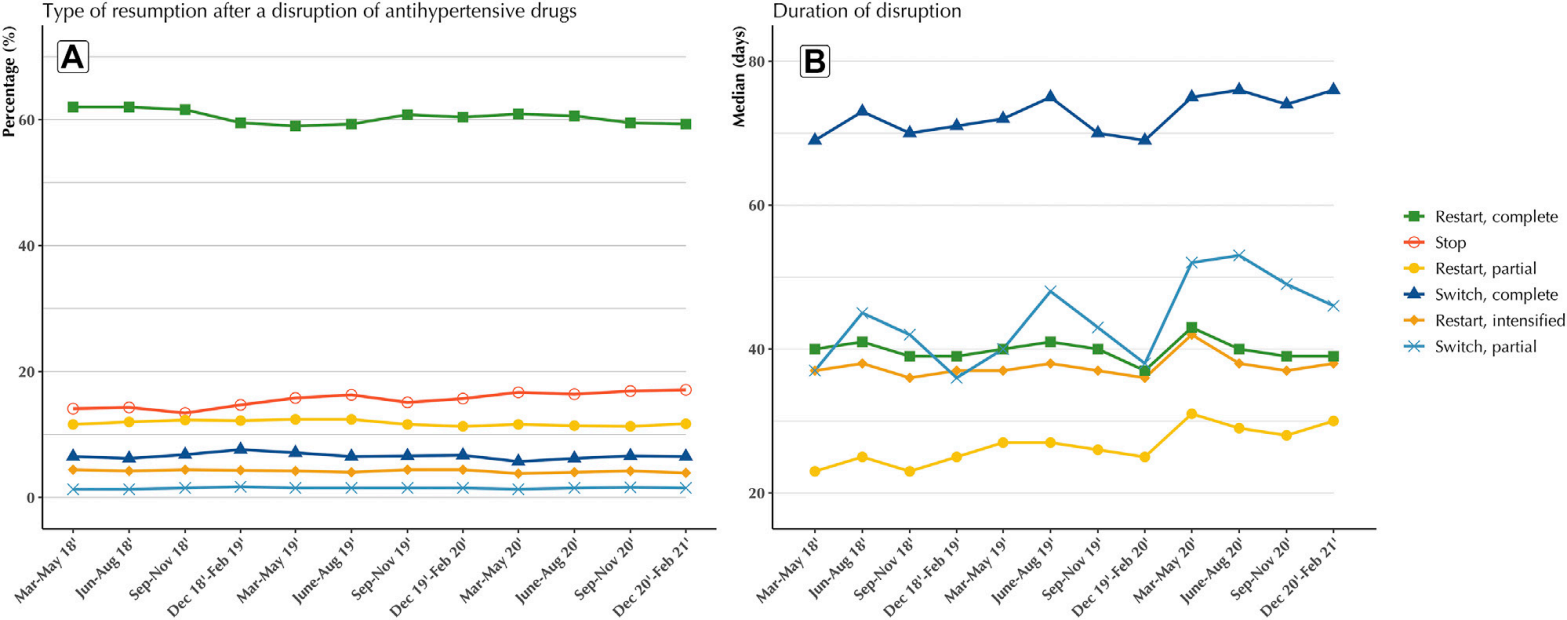
Evolution of the distribution of antihypertensive drug treatment outcomes for the disruptions observed between March 2018 and February 2021 in France **(A)**, and evolution of the median duration before treatment resumption **(B)**.

No impact of the COVID-19 outbreak was evident on treatment outcome at 1 year after a disruption (Figure 4; Table 2). The proportion of patients who fully resumed treatment after a disruption showed a steady relative decrease of 6.7% throughout the period from March–May 2018 to December 2020–February 2021 [62.0%–58.1%; main trend: 0.30 (−0.58; −0.01)], whereas the 1-year dropout rate increased relatively by 29.8% over the period (14.1%–18.3%), also at a steady rate [main trend: 0.30 (0.09; 0.50)]. This change was independent of any effect of the COVID-19 epidemic, with no significant change in level or trend after the COVID-19 epidemic evident for the proportion of complete treatment resumption [COVID-19 epidemic level change: 2.66 (−0.11; 5.42); slope change: −0.67 (−1.54; 0.20)] or for that of dropout [change in COVID-19 epidemic level: −0.21 (−2.08; 1.65); change in slope: 0.24 (−0.40; 0.87)]. Similarly, no significant impact was observed on switches (Table 2).

**TABLE 2:** Results of the interrupted time-series quarterly analysis for the effect of COVID epidemic on the rates of disruption of antihypertensive drugs between March 2018, and February 2021, expressed in Rate ratios considering the March to May quarter as the epidemic breakpoint date.

	Restart, complete	Restart, intensified	Restart, partial	Switch, partial	Switch, complete	Stop
Slope	−0.30 (−0.58; −0.01)*	0.00 (−0.05; 0.05)	−0.04 (−0.16; 0.08)	0.02 (−0.01; 0.05)	0.02 (−0.11; 0.14)	0.30 (0.09; 0.50)*
Level change of COVID-epidemic	2.66 (−0.11; 5.42)	−0.44 (−0.88; 0.00)	−0.45 (−1.52; 0.62)	−0.26 (−0.54; 0.02)	−1.29 (−2.69; 0.11)	−0.21 (−2.08; 1.65)
Slope change of COVID-epidemic	−0.67 (−1.54; 0.20)	0.05 (−0.10; 0.20)	0.06 (−0.31; 0.42)	0.05 (−0.04; 0.15)	0.27 (−0.11; 0.66)	0.24 (−0.40; 0.87)

***p < 0.001; **p < 0.01; *p < 0.05.

Regardless of the treatment restart model, the epidemic did not appear to alter the time to restart after a disruption (Figure 4). Although a slight spike was observed during the March-May 2020 period, the median time to restart was constant over the entire period and approximately 35–40 days for Full or Intensified Restart), constant. For the other modalities (Partial Restart, Switches complete or partial), while an increasing trend was observed over the entire period, no impact of the pandemic was found for the time from treatment disruption to restart. Similarly, additional analyses assessing the impact of the COVID-19 epidemic on treatment outcome after discontinuation, including by individual pharmacological class of antihypertensive drugs, showed no variation during the epidemic in either the rate of resumption after discontinuation or the duration of discontinuation. All estimates of the number of antihypertensive drug disruptions, as well as estimates of the rates of resumption after disruption, and the duration of disruption, are detailed in the Supplementary Material.

## Discussion

The epidemic of COVID-19 has disrupted the management of patients: overcrowding of emergency services, long and repeated lockdowns (Cauchemez et al., 2020; Spaccaferri et al., 2020; Cransac-Miet et al., 2021), stress induced by the spread of the virus (Moreno et al., 2020; Salari et al., 2020). Like others, we have previously highlighted that in France, the first lockdown led to prescription stoppages (Levaillant et al., 2021; Mathieu et al., 2022; Sanchez et al., 2022) and was accompanied by an excess of cardiovascular drug treatment shortages (Mathieu et al., 2022). In addition, we were concerned that the COVID-19 epidemic may have had a more profound impact on cardiovascular treatment disruptions, either by worsening their characteristics or by causing them in patients who would usually have been at lower risk. In the study we report here, we were able to complement these findings by specifically exploring disruptions that involved antihypertensive treatments. For these treatments, disruptions that occurred after the onset of the pandemic, even in excess, showed no specificity in terms of patient characteristics or treatment outcome after the disruption compared with treatment disruptions studied over the 2-year period before the pandemic. The results were consistent for each class of antihypertensive drugs considered individually and controlling for patient age and sex. Indeed, we found no significant impact of the COVID-19 epidemic either on the rate of resumption or change of treatment after disruption or on the time between disruption and restart of treatment after disruption, regardless of the modality.

Thus, this study provided reassuring results, showing, for antihypertensive drugs, no impact on treatment discontinuations other than the quantitative excess already discussed. The median duration of treatment discontinuation remained unchanged over the entire period and was similar between the pre-COVID and COVID eras. However, it was far from zero, with, for example, a median time of 40 days between treatment disruption and full resumption. Since the outcome of treatment after disruption was also not affected by the epidemic, the individual consequences of epidemic-induced disruptions should therefore be similar to those normally observed.

Despite this, the incidence of death within 1 month or 1 year of treatment disruption appeared potentially higher for disruptions occurring in the quarter after the start of the COVID-19 epidemic than for those occurring in the corresponding quarters of 2018 and 2019. Because the characteristics of the disruptions did not differ between periods, the most plausible explanation for this potential difference could lie in the overall excess mortality associated with the pandemic (Insee, 2022). However, this hypothesis would need to be explored further because this study does not rule out a difference in the effect on health outcomes of COVID-induced disruptions compared with pre-COVID disruptions. The latter could be related not to the disruptions *per se* but to the potential changes that the epidemic induced in the management of major adverse cardiovascular events during the period because of the disorganization of care.

Second, although there was no effect of the COVID-19 epidemic on treatment outcome after discontinuation, his study found evidence throughout the study period of a steady decrease in treatment resumption and a parallel steady increase in discontinuation. This appears to be of great concern, potentially more so than if it were related to the COVID-19 epidemic, as the results presented here point to a deeper trend of change. These results are consistent with the favourable hypothesis of a trend toward population deprescribing; they would then reflect the therapeutic decision of prescribers and the resulting tailored treatment changes. However, they are also compatible with the alternative hypothesis of a progressive deterioration in compliance with antihypertensive treatment. The potential consequences of this hypothesis also invite further investigation of these results. This would require more clinical information than is available in the database we used, and ideally information on why treatment was discontinued.

The distribution of the deprivation index showed an increased trend, individuals residing in the richest locations being the less represented in those who interrupted an antihypertensive drug, and those leaving in the poorest ones being the most. This potentially reflects a higher susceptibility of those residing in the poorest areas to experience an antihypertensive drug disruption already reported in the literature (Kirschbaum et al., 2022). The data we present cannot however fully support this hypothesis as we only analysed data on patients with antihypertensive drug interruptions; it could indeed only be that all antihypertensive drug users were more likely to reside in deprived areas.

This study focuses on a topic that is not widely discussed in the literature yet. However, if the impact of the COVID-19 epidemic on interruptions of antihypertensive treatment has been little assessed, several studies have shown a worrying decrease in the use of chronic drugs over the pandemic, in fields as various as mental disorders (Mansfield et al., 2021; Sanchez et al., 2022) or diabetes for instance (Corral-Partearroyo et al., 2022; Mathieu et al., 2022). Considering more specifically cardiovascular drugs, our results appear consistent with those of a study assessing the impact of the epidemic on adherence to statins (Malo et al., 2022).

This study has some important strengths. First, the use of the main affiliation database of the SNDS allows us to provide a national evaluation obtained from data concerning almost 90% of the total French population. In addition, this database allows us to analyse exhaustive information on outpatient drug dispensing except for those obtained over the counter; the dispensing information we used for antihypertensives, which all require a medical prescription and are reimbursed for all affiliates, is therefore likely to be complete. Similarly, age and sex had no missing data in the database. Information on inpatient diagnoses is also complete for all hospital stays but was not used for the assessment of comorbidities in this study. Contrary to what we usually do when we carry out studies using the SNDS, we preferred to consider here the information from the Affections de Longue Durée because the stability of the hospital coding information could not be guaranteed over the study period. This potential variability in coding completeness results from a coding strike among Parisian hospital practitioners that lasted from mid-October 2019 to mid-February 2020 (Agence technique de l’information sur l’hospitalisation, 2019). A limitation of the study we report, as with all studies using claims databases, is that it cannot provide information on patients’ actual adherence to their treatment. The information available is in fact only dispensing information, allowing us to consider that the patient to whom a drug was prescribed was actually in possession of it; the assumption is made that he or she used it, but this cannot be fully confirmed. Second, drug consumption in hospitals is not available in the French health insurance databases, which leads to censoring of drug consumption information (Bezin et al., 2017). The resulting immeasurable temporal bias consists of a measurement error where treatments continued during hospital stays are erroneously considered interrupted when the continuity of treatment is assessed from information on outpatient drug dispensing, as was the case in the study we report (Suissa, 2008). However, for such a bias to occur, censoring must be differential between the groups or time period being compared in a way that contributes to the conclusion. This was not the case here, with the rate of hospitalization prior to discontinuation remaining stable or at worst decreasing slightly over this period (Direction de la recherche et al., 2021). If it existed, the resulting potential bias should therefore have been limited and should have led to a decrease in the erroneous overestimation of the treatment discontinuation rate during the COVID period compared with the pre-COVID period. The excess treatment disruption results presented here can therefore be considered conservative if affected by this potential censoring, as discussed previously (Mathieu et al., 2022).

## Conclusion

This study showed that, although it led to an increase in cardiovascular treatment discontinuations, the COVID-19 epidemic did not change the characteristics of these. First, breaks were not prolonged and treatment outcomes after breaks remained unchanged. Second, patients who experienced a break in antihypertensive therapy during the COVID-19 outbreak were essentially similar to those who experienced breaks before it. This study, however, provided secondary findings that require further investigation. First, a potentially higher mortality rate after the COVID-19 epidemic disruptions but also related to deaths during the COVID-19 epidemic. Second, a consistent decrease in the rate of treatment resumption after disruption over the independent period of the COVID-19 epidemic, which may reflect a gradual deterioration in adherence to antihypertensive treatment.

## Consent to participation and publication

In accordance with regulations, the study was authorized by the *Commission nationale de l’informatique et des libertés* and by the *Institut français des données de santé*.

No patient was involved in defining the research question or outcome measures, or in developing the study design or implementation plans. No patients were asked to provide input into the interpretation or writing of the results. There are no plans to disseminate the results of the research to study participants or the relevant patient community.

## Data Availability

The data analyzed in this study is subject to the following licenses/restrictions: data from the French administrative healthcare databases (SNDS) were on the SNDS portal. Requests to access these datasets should be directed to https://www.health-data-hub.fr/.
