# Supplementary material for: Impact of COVID-19 epidemic on antihypertensive drug treatment disruptions: results from a nationwide interrupted time-series analysis
Source: Front Pharmacol. 2023 May 15;14:1129244. doi: 10.3389/fphar.2023.1129244 (PMC10225585; doi:10.3389/fphar.2023.1129244)
Supplement: Supplementary file 1 [file DataSheet2.pdf]

Annex 3 – Table 1. Rate ratios for the different types of resumption for patients interrupting antihypertensive drugs categories of interest between March, 2018, and February, 2021, among French National Health Data System, considering that the epidemic breakpoint date was March, 2020.

		**Restart, complete**	**Restart, intensified**	**Restart, partial**	**Switch, partial**	**Switch, complete**	**Stop**
**Antihypertensive drugs**	Time	-0.30 [-0.58; -0.01]	0.00 [-0.05; 0.05]	-0.04 [-0.16; 0.08]	0.02 [-0.01; 0.05]	0.02 [-0.11; 0.14]	0.30 [0.09; 0.50]
	Covid	2.66 [-0.11; 5.42]	-0.44 [-0.88; 0.00]	-0.45 [-1.52; 0.62]	-0.26 [-0.54; 0.02]	-1.29 [-2.69; 0.11]	-0.21 [-2.08; 1.65]
	Time_after	-0.67 [-1.54; 0.20]	0.05 [-0.10; 0.20]	0.06 [-0.31; 0.42]	0.05 [-0.04; 0.15]	0.27 [-0.11; 0.66]	0.24 [-0.40; 0.87]
**Treatment incl. Beta-blockers**	Time	-0.30 [-0.43; -0.17]	0.00 [-0.04; 0.04]	-0.08 [-0.18; 0.02]	0.01 [-0.02; 0.04]	0.00 [-0.03; 0.02]	0.36 [0.21; 0.52]
	Covid	0.19 [-0.99; 1.38]	-0.42 [-0.87; 0.04]	-0.35 [-1.25; 0.55]	-0.23 [-0.47; 0.00]	-0.29 [-0.52; 0.03]	1.2 [-0.20; 2.60]
	Time_after	0.07 [-0.33; 0.48]	0.04 [-0.07; 0.16]	0.08 [-0.23; 0.39]	0.06 [-0.02; 0.14]	0.11 [0.04; 0.18]	-0.39 [-0.87; 0.08]
**Treatment incl. ACE inhibitors**	Time	-0.28 [-0.45; -0.11]	-0.02 [-0.06; 0.02]	-0.12 [-0.24; -0.01]	0.03 [0.01; 0.05]	0.02 [-0.01; 0.04]	0.37 [0.22; 0.52]
	Covid	1.97 [-0.01; 3.95]	-0.47 [-0.99; 0.05]	-0.86 [-1.87; 0.15]	-0.32 [-0.70; 0.06]	-0.39 [-0.81; 0.03]	0.04 [-1.26; 1.35]
	Time_after	-0.44 [-0.96; 0.08]	0.07 [-0.06; 0.19]	0.41 [0.07; 0.76]	0.04 [-0.01; 0.09]	0.03 [-0.05; 0.11]	-0.11 [-0.56; 0.34]
**Treatment incl. ARBs**	Time	-0.25 [-0.54; 0.04]	0.03 [-0.01; 0.06]	-0.11 [-0.27; 0.05]	-0.02 [-0.08; 0.05]	-0.04 [-0.14; 0.06]	0.39 [0.26; 0.51]
	Covid	1.53 [-1.04; 4.09]	-0.53 [-1.06; 0.01]	-0.57 [-1.99; 0.86]	-0.23 [-0.79; 0.32]	-0.46 [-1.38; 0.46]	0.23 [-0.88; 1.33]
	Time_after	-0.42 [-1.30; 0.46]	0.10 [0.00; 0.19]	0.33 [-0.16; 0.82]	0.06 [-0.13; 0.24]	0.15 [-0.16; 0.46]	-0.18 [-0.55; 0.20]
**Treatment incl. CCBs**	Time	-0.25 [-0.44; -0.05]	0.04 [0.01; 0.07]	-0.17 [-0.29; -0.05]	0.01 [-0.04; 0.05]	0.01 [-0.02; 0.04]	0.38 [0.20; 0.55]
	Covid	1.51 [-0.27; 3.28]	-0.53 [-1.08; 0.02]	-0.35 [-1.45; 0.75]	-0.26 [-0.65; 0.14]	-0.25 [-0.51; 0.01]	-0.09 [-1.64; 1.45]
	Time_after	-0.3 [-0.91; 0.30]	0.01 [-0.09; 0.11]	0.15 [-0.22; 0.53]	0.06 [-0.07; 0.20]	0.06 [-0.03; 0.15]	-0.01 [-0.53; 0.52]
**Treatment incl. Thiazide**	Time	-0.49 [-0.94; -0.03]	0.02 [-0.02; 0.07]	0.03 [-0.27; 0.33]	0.04 [-0.01; 0.10]	0.01 [-0.02; 0.04]	0.38 [0.18; 0.59]
	Covid	2.95 [-1.17; 7.07]	-0.33 [-0.75; 0.09]	-1.58 [-4.26; 1.11]	-0.44 [-0.95; 0.07]	-0.19 [-0.48; 0.09]	-0.36 [-2.19; 1.47]
	Time_after	-0.18 [-1.59; 1.22]	0.03 [-0.12; 0.17]	0.12 [-0.80; 1.04]	0.07 [-0.11; 0.24]	0.03 [-0.07; 0.13]	-0.08 [-0.71; 0.55]

Annex 3 – Table 2. Rate ratios for the different types of resumption for patients interrupting only one of antihypertensive drugs categories of interest between March 2018 and February 2021 among French National Health Data System considering that the epidemic breakpoint date was March. 2020.

		**Restart, complete**	**Restart, intensified**	**Switch, complete**	**Stop**
**Beta-blockers**	Time	-0.63 [-0.80; -0.45]	-0.06 [-0.13; 0.01]	-0.01 [-0.06; 0.04]	0.70 [0.52; 0.88]
	Covid	-0.33 [-1.89; 1.24]	-0.51 [-1.13; 0.11]	-0.69 [-1.40; 0.01]	1.59 [-0.04; 3.23]
	Time_after	0.20 [-0.34; 0.73]	0.05 [-0.16; 0.26]	0.23 [0.08; 0.39]	-0.51 [-1.07; 0.05]
**ACE inhibitors**	Time	-0.43 [-0.61; -0.24]	-0.08 [-0.15; -0.01]	0.00 [-0.06; 0.06]	0.46 [0.27; 0.64]
	Covid	1.23 [-0.44; 2.90]	-0.68 [-1.38; 0.02]	-0.98 [-1.96; 0.01]	0.25 [-1.05; 1.55]
	Time_after	0.04 [-0.53; 0.61]	0.18 [-0.03; 0.39]	0.14 [-0.05; 0.32]	-0.03 [-0.47; 0.42]
**ARBs**	Time	-0.35 [-0.61; -0.10]	0.02 [-0.04; 0.08]	-0.12 [-0.38; 0.13]	0.52 [0.31; 0.73]
	Covid	1.36 [-0.95; 3.67]	-0.53 [-1.08; 0.01]	-1.02 [-3.29; 1.25]	0.93 [-0.98; 2.83]
	Time_after	-0.41 [-1.20; 0.38]	0.08 [-0.10; 0.26]	0.35 [-0.42; 1.13]	-0.59 [-1.24; 0.06]
**CCBs**	Time	-0.63 [-0.94; -0.33]	0.01 [-0.07; 0.08]	-0.04 [-0.13; 0.05]	0.49 [0.31; 0.67]
	Covid	-0.07 [-2.79; 2.66]	-0.80 [-1.61; 0.01]	-0.51 [-1.31; 0.29]	0.49 [-1.11; 2.09]
	Time_after	0.83 [-0.10; 1.77]	0.04 [-0.19; 0.27]	0.16 [-0.11; 0.43]	-0.36 [-0.91; 0.18]
**Thiazide**	Time	-0.93 [-1.27; -0.59]	0.28 [0.18; 0.38]	0.14 [-0.02; 0.30]	0.68 [0.42; 0.94]
	Covid	2.82 [-0.23; 5.86]	-1.83 [-3.70; 0.03]	-1.93 [-3.88; 0.01]	1.20 [-1.15; 3.55]
	Time_after	0.66 [-0.38; 1.70]	-0.32 [-0.64; 0.01]	0.24 [-0.24; 0.73]	-1.02 [-1.82; -0.22]

Annex 3 – Table 3. Rate ratios for the different types of resumption for patients interrupting antihypertensive drugs, by sex or age, between March, 2018, and February, 2021, among French National Health Data System, considering that the epidemic breakpoint date was March, 2020.

		**Restart, complete**	**Restart, intensified**	**Restart, partial**	**Switch, partial**	**Switch, complete**	**Stop**
**Men**	Time	-0,34 [-0,65; -0,02]	0,00 [-0,05; 0,06]	-0,04 [-0,20; 0,12]	0,02 [-0,01; 0,05]	0,03 [-0,10; 0,16]	0,33 [0,12; 0,54]
	Covid	3,04 [0,20; 5,89]	-0,52 [-1,01; -0,03]	-0,86 [-2,28; 0,56]	-0,24 [-0,48; 0,00]	-1,29 [-2,42; -0,16]	-0,17 [-2,01; 1,67]
	Time_after	-0,68 [-1,66; 0,29]	0,07 [-0,10; 0,24]	0,27 [-0,22; 0,76]	0,05 [-0,03; 0,13]	0,25 [-0,13; 0,64]	0,04 [-0,59; 0,67]
**Women**	Time	-0,25 [-0,51; 0,01]	0,00 [-0,04; 0,05]	-0,03 [-0,13; 0,06]	0,03 [-0,01; 0,06]	0,01 [-0,12; 0,15]	0,25 [0,06; 0,45]
	Covid	2,28 [-0,04; 4,59]	-0,46 [-0,88; -0,04]	-0,59 [-1,46; 0,27]	-0,32 [-0,61; -0,02]	-1,27 [-2,46; -0,07]	0,31 [-1,44; 2,06]
	Time_after	-0,69 [-1,48; 0,10]	0,04 [-0,11; 0,18]	0,17 [-0,12; 0,47]	0,07 [-0,03; 0,18]	0,27 [-0,14; 0,68]	0,12 [-0,48; 0,71]
**Under 65 Years old**	Time	-0,41 [-0,71; -0,12]	0,01 [-0,03; 0,05]	-0,02 [-0,10; 0,05]	0,01 [-0,02; 0,04]	0,02 [-0,09; 0,13]	0,39 [0,16; 0,63]
	Covid	1,68 [-0,95; 4,31]	-0,31 [-0,67; 0,06]	-0,37 [-1,06; 0,32]	-0,08 [-0,32; 0,15]	-0,99 [-1,98; -0,01]	0,18 [-1,91; 2,26]
	Time_after	-0,33 [-1,23; 0,57]	0,03 [-0,10; 0,15]	0,06 [-0,17; 0,30]	0,02 [-0,06; 0,10]	0,2 [-0,13; 0,54]	0,01 [-0,71; 0,72]
**At least 65 years old**	Time	-0,29 [-0,58; -0,01]	0,00 [-0,06; 0,05]	0,00 [-0,15; 0,15]	0,03 [-0,01; 0,06]	0,03 [-0,1; 0,16]	0,23 [0,06; 0,40]
	Covid	2,27 [-0,26; 4,79]	-0,46 [-0,95; 0,04]	-0,53 [-1,87; 0,81]	-0,23 [-0,56; 0,09]	-1,39 [-2,58; -0,20]	0,49 [-1,01; 1,99]
	Time_after	-0,35 [-1,21; 0,52]	0,04 [-0,12; 0,21]	0,15 [-0,31; 0,61]	0,05 [-0,06; 0,16]	0,31 [-0,10; 0,72]	-0,22 [-0,73; 0,29]
