# Supplementary material for: Impact of COVID-19 epidemic on antihypertensive drug treatment disruptions: results from a nationwide interrupted time-series analysis
Source: Front Pharmacol. 2023 May 15;14:1129244. doi: 10.3389/fphar.2023.1129244 (PMC10225585; doi:10.3389/fphar.2023.1129244)
Supplement: Supplementary file 2 [file Table1.PDF]

Supplementary material 1. ATC codes used to identify antihypertensive drug categories

**Drug categories**	**ATC Codes**
Angiotensin-converting-enzyme inhibitors (ACE inhibitors)	C09A: ACE inhibitors, plain
	C09B: ACE inhibitors, combinations
Beta-blockers	C07: Beta blocking agents
Angiotensin II receptor blockers (ARBs)	C09C: ARBs, plain
	C09D: ARBs, combinations
Calcium channel blockers (CCBs)	C08: Calcium channel blockers
	C09BB: ACE inhibitors and calcium channel blockers
	C09DB: ARBs and calcium channel blockers
	C10BX03: Atorvastatin and amlodipine
Thiazide	C03A: low-ceiling diuretics, thiazides
	C03B: Low-ceiling diuretics, excluding thiazides
	C02LA01: Reserpine and diuretics
	C03EA: Low-ceiling diuretics and potassium-sparing agents
	C07B: Beta blocking agents and thiazides
	C07C: Beta blocking agents and other diuretics
	C07D: Beta blocking agents, thiazides and other diuretics
	C08G: Calcium channel blockers and diuretics
	C09BA: ACE inhibitors and diuretics
	C09DA: ARBs and diuretics
	C09DX01: valsartan, amlodipine and hydrochlorothiazide
	C09XA52: aliskiren and hydrochlorothiazide
Other antihypertensive drugs	C03D: Aldosterone antagonists and other potassium-sparing agents
	C03E: Diuretics and potassium-sparing agents in combination
	C03C: High-ceiling diuretics
	C03EB: High-ceiling diuretics and potassium-sparing agents
	C02A: Antiadrenergic agents, centrally acting
	C02CA: Alpha-adrenoreceptor antagonists
	C02DC01: minoxidil
