# Supplementary material for: Impact of COVID-19 epidemic on antihypertensive drug treatment disruptions: results from a nationwide interrupted time-series analysis
Source: Front Pharmacol. 2023 May 15;14:1129244. doi: 10.3389/fphar.2023.1129244 (PMC10225585; doi:10.3389/fphar.2023.1129244)
Supplement: Supplementary file 3 [file DataSheet1.pdf]

Annex 2 - Table 1. Distribution of treatment disruptions in patients using only one antihypertensive drug class.

		Mar 18' - May 18'	Jun 18' - Aug 18'	Sep 18' - Nov 18'	Dec 18' - Feb 19'	Mar 19' - May 19'	Jun 19' - Aug 19'	Sep 19' - Nov 19'	Dec 19' - Feb 20'	Mar 20' - May 20'	Jun 20' - Aug 20'	Sep 20' - Nov 20'	Dec 20' - Feb 21'
**Beta-blockers**	Restart, complete	185595 (75.1)	197559 (75.5)	191494 (74.9)	185356 (73.4)	187651 (72.7)	189998 (72.4)	185750 (72.1)	173757 (71.2)	191229 (70.7)	182438 (70.2)	165492 (68.6)	173846 (69.8)
	Restart, intensified	15533 (6.3)	15975 (6.1)	16495 (6.4)	15796 (6.3)	15035 (5.8)	14875 (5.7)	15476 (6.0)	14600 (6.0)	13949 (5.2)	14058 (5.4)	13398 (5.6)	12673 (5.1)
	Switch, complete	12484 (5.1)	12899 (4.9)	13532 (5.3)	13423 (5.3)	13092 (5.1)	12743 (4.9)	13120 (5.1)	12161 (5.0)	12141 (4.5)	12554 (4.8)	12559 (5.2)	12598 (5.1)
	Stop	33530 (13.6)	35095 (13.4)	34260 (13.4)	37862 (15)	42506 (16.5)	44847 (17.1)	43205 (16.8)	43544 (17.8)	53151 (19.7)	50859 (19.6)	49769 (20.6)	49932 (20.0)
**ACE inhibitors**	Restart, complete	111977 (76.0)	116340 (76.7)	118353 (76.3)	110248 (75.3)	113497 (74)	119355 (74.3)	117355 (74.2)	107997 (73.7)	121877 (74.6)	120411 (74.2)	106830 (72.7)	110100 (73.8)
	Restart, intensified	10408 (7.1)	10130 (6.7)	10736 (6.9)	10007 (6.8)	10011 (6.5)	9828 (6.1)	10370 (6.6)	9668 (6.6)	9294 (5.7)	9712 (6.0)	8989 (6.1)	8872 (6.0)
	Switch, complete	10423 (7.1)	10252 (6.8)	11252 (7.3)	10673 (7.3)	11127 (7.3)	11152 (6.9)	11197 (7.1)	10339 (7.1)	10213 (6.3)	10149 (6.3)	10037 (6.8)	9787 (6.6)
	Stop	14607 (9.9)	15055 (9.9)	14812 (9.5)	15447 (10.6)	18682 (12.2)	20311 (12.6)	19268 (12.2)	18534 (12.6)	21895 (13.4)	22025 (13.6)	20993 (14.3)	20341 (13.6)
**ARBs**	Restart, complete	154382 (81.4)	156357 (80.9)	168868 (80.1)	152134 (78.1)	152706 (78.2)	157359 (78.9)	165837 (79.6)	147525 (78.6)	162946 (78.9)	160209 (78.2)	142207 (76.9)	142094 (76.8)
	Restart, intensified	10788 (5.7)	10376 (5.4)	11240 (5.3)	10975 (5.6)	11287 (5.8)	10812 (5.4)	11417 (5.5)	10808 (5.8)	10666 (5.2)	10727 (5.2)	10353 (5.6)	9978 (5.4)
	Switch, complete	8853 (4.7)	9991 (5.2)	13524 (6.4)	13901 (7.1)	10723 (5.5)	9341 (4.7)	9553 (4.6)	8654 (4.6)	8462 (4.1)	8896 (4.3)	8880 (4.8)	8626 (4.7)
	Stop	15633 (8.2)	16502 (8.5)	17265 (8.2)	17709 (9.1)	20541 (10.5)	21977 (11)	21492 (10.3)	20725 (11)	24558 (11.9)	25123 (12.3)	23471 (12.7)	24379 (13.2)
**CCBs**	Restart, complete	94441 (71.8)	96690 (72.7)	97890 (72.8)	91999 (70.9)	95270 (68.7)	95986 (69.1)	96838 (69.6)	90362 (68.3)	101451 (68.9)	98691 (68.4)	87876 (67.7)	92461 (69.8)
	Restart, intensified	10757 (8.2)	10525 (7.9)	11267 (8.4)	10900 (8.4)	11220 (8.1)	10897 (7.8)	11517 (8.3)	10921 (8.3)	10770 (7.3)	10978 (7.6)	10114 (7.8)	9761 (7.4)
	Switch, complete	11485 (8.7)	11027 (8.3)	11184 (8.3)	11244 (8.7)	12145 (8.8)	11502 (8.3)	11552 (8.3)	10828 (8.2)	11206 (7.6)	12114 (8.4)	10837 (8.4)	10567 (8.0)
	Stop	14823 (11.3)	14787 (11.1)	14044 (10.5)	15546 (12.0)	19962 (14.4)	20524 (14.8)	19302 (13.9)	20241 (15.3)	23769 (16.1)	22531 (15.6)	20921 (16.1)	19620 (14.8)
**Thiazide**	Restart, complete	13404 (64.6)	13765 (64.4)	13809 (63.3)	13131 (62.7)	13223 (59.7)	13150 (59.5)	13606 (58.6)	12672 (59.6)	14169 (61.5)	13479 (60.4)	11993 (58.6)	12666 (61.2)
	Restart, intensified	2289 (11.0)	2303 (10.8)	2466 (11.3)	2404 (11.5)	2755 (12.3)	2605 (11.8)	3029 (12.9)	2645 (12.5)	2412 (10.7)	2443 (10.9)	2296 (11.1)	2172 (10.5)
	Switch, complete	2435 (11.7)	2603 (12.2)	2902 (13.2)	2735 (13.1)	2932 (13.2)	2914 (13.2)	3045 (13.1)	2675 (12.7)	2592 (11.4)	2786 (12.3)	2716 (13.1)	2559 (12.4)
	Stop	2624 (12.6)	2719 (12.7)	2647 (12.2)	2685 (12.8)	3319 (14.8)	3425 (15.5)	3555 (15.3)	3237 (15.3)	3803 (16.4)	3677 (16.4)	3557 (17.2)	3296 (15.9)

Annex 2 - Table 2. Distribution of treatment disruptions in patients using several antihypertensive drug classes

		**Mar 18' - May 18'**	**Jun 18' - Aug 18'**	**Sep 18' - Nov 18'**	**Dec 18' - Feb 19'**	**Mar 19' - May 19'**	**Jun 19' - Aug 19'**	**Sep 19' - Nov 19'**	**Dec 19' - Feb 20'**	**Mar 20' - May 20'**	**Jun 20' - Aug 20'**	**Sep 20' - Nov 20'**	**Dec 20' - Feb 21'**
**Treatment incl. Beta-blockers**	Restart, complete	433326 (61.3)	458475 (61.3)	447527 (60.7)	426673 (59.8)	430386 (59.3)	437714 (59.3)	434413 (59.7)	405290 (59.5)	449567 (59.0)	432703 (59.0)	391267 (58.2)	389629 (58.5)
	Restart, intensified	26422 (3.7)	27453 (3.7)	28729 (3.9)	27384 (3.8)	26285 (3.6)	25942 (3.5)	27476 (3.8)	26014 (3.8)	25212 (3.3)	25107 (3.4)	23837 (3.5)	22979 (3.4)
	Restart, partial	121584 (17.2)	129669 (17.3)	132151 (17.9)	125383 (17.6)	127074 (17.5)	128119 (17.3)	125007 (17.2)	113003 (16.6)	128070 (16.8)	122370 (16.7)	110526 (16.4)	112778 (16.9)
	Switch, complete	15222 (2.2)	15777 (2.1)	16618 (2.3)	16473 (2.3)	16173 (2.2)	15709 (2.1)	16058 (2.2)	14833 (2.2)	14933 (2.0)	15436 (2.1)	15441 (2.3)	15492 (2.3)
	Switch, partial	8861 (1.3)	8932 (1.2)	10209 (1.4)	10499 (1.5)	10048 (1.4)	9584 (1.3)	9791 (1.3)	9203 (1.4)	9072 (1.2)	9234 (1.3)	9387 (1.4)	9494 (1.4)
	Stop	101990 (14.4)	107918 (14.4)	101614 (13.8)	106546 (14.9)	116157 (16)	121408 (16.4)	115183 (15.8)	112287 (16.5)	134558 (17.7)	127939 (17.5)	121505 (18.1)	115964 (17.4)
**Treatment incl. ACE inhibitors**	Restart, complete	318099 (66.2)	333727 (66.5)	339917 (66.6)	318420 (65.4)	330270 (64.5)	339647 (64.6)	340321 (65.0)	316945 (64.9)	356744 (65.3)	353372 (65.5)	318968 (64.6)	314357 (63.2)
	Restart, intensified	20353 (4.2)	20355 (4.1)	21714 (4.3)	20287 (4.2)	20359 (4.0)	20295 (3.9)	21567 (4.1)	20358 (4.2)	19665 (3.6)	20205 (3.7)	19117 (3.9)	18531 (3.7)
	Restart, partial	70091 (14.6)	72680 (14.5)	73748 (14.5)	71590 (14.7)	74696 (14.6)	74922 (14.2)	72818 (13.9)	66673 (13.7)	74746 (13.7)	72551 (13.4)	65643 (13.3)	73016 (14.7)
	Switch, complete	13484 (2.8)	13575 (2.7)	14841 (2.9)	14538 (3.0)	15070 (2.9)	15137 (2.9)	15043 (2.9)	13982 (2.9)	14093 (2.6)	14176 (2.6)	13912 (2.8)	13646 (2.7)
	Switch, partial	6557 (1.4)	6641 (1.3)	7170 (1.4)	7264 (1.5)	7789 (1.5)	7796 (1.5)	8238 (1.6)	7507 (1.5)	7237 (1.3)	7397 (1.4)	7331 (1.5)	7347 (1.5)
	Stop	52027 (10.8)	54545 (10.9)	52866 (10.4)	54713 (11.2)	63833 (12.5)	68148 (13)	65363 (12.5)	62677 (12.8)	73578 (13.5)	72158 (13.4)	68696 (13.9)	70493 (14.2)
**Treatment incl. ARBs**	Restart, complete	419336 (69.8)	426009 (68.8)	451465 (67.7)	407644 (66.2)	407983 (66.8)	418864 (67.2)	445635 (68.0)	407957 (67.5)	452799 (67.3)	438864 (67.5)	396666 (66.8)	388893 (65.3)
	Restart, intensified	24549 (4.1)	23938 (3.9)	26174 (3.9)	24774 (4.0)	25204 (4.1)	24789 (4.0)	26636 (4.1)	25385 (4.2)	25064 (3.7)	24787 (3.8)	24257 (4.1)	23657 (4.0)
	Restart, partial	81416 (13.6)	88783 (14.3)	99493 (14.9)	89076 (14.5)	85138 (13.9)	86661 (13.9)	88909 (13.6)	80199 (13.3)	91321 (13.6)	86201 (13.3)	77909 (13.1)	85554 (14.4)
	Switch, complete	12778 (2.1)	14395 (2.3)	18636 (2.8)	19086 (3.1)	15414 (2.5)	13707 (2.2)	14045 (2.1)	12708 (2.1)	12710 (1.9)	13207 (2.0)	13130 (2.2)	12915 (2.2)
	Switch, partial	6993 (1.2)	7461 (1.2)	9742 (1.5)	11033 (1.8)	8528 (1.4)	7404 (1.2)	7679 (1.2)	7298 (1.2)	7180 (1.1)	7238 (1.1)	7126 (1.2)	7190 (1.2)
	Stop	55727 (9.3)	58192 (9.4)	61089 (9.2)	64410 (10.5)	68264 (11.2)	71845 (11.5)	72058 (11)	70491 (11.7)	83527 (12.4)	79897 (12.3)	74993 (12.6)	77384 (13.0)
**Treatment incl. CCBs**	Restart, complete	337462 (61.6)	347932 (61.4)	353403 (61.2)	326932 (60.1)	336049 (59.2)	341189 (59.4)	349618 (60.5)	327466 (60.2)	369767 (60.3)	364191 (60.2)	327665 (59.8)	325176 (58.6)
	Restart, intensified	22605 (4.1)	22609 (4.0)	24329 (4.2)	23249 (4.3)	23564 (4.2)	23336 (4.1)	25061 (4.3)	23726 (4.4)	23161 (3.8)	23626 (3.9)	22240 (4.1)	21597 (3.9)
	Restart, partial	96774 (17.7)	102216 (18.0)	106950 (18.5)	98548 (18.1)	101202 (17.8)	101600 (17.7)	99350 (17.2)	90473 (16.6)	103559 (16.9)	100382 (16.6)	89886 (16.4)	93815 (16.9)
	Switch, complete	15055 (2.7)	14924 (2.6)	15401 (2.7)	15310 (2.8)	16439 (2.9)	15728 (2.7)	15721 (2.7)	14773 (2.7)	15302 (2.5)	16585 (2.7)	15136 (2.8)	14831 (2.7)
	Switch, partial	9383 (1.7)	9347 (1.6)	10402 (1.8)	11290 (2.1)	10747 (1.9)	10022 (1.7)	10123 (1.8)	9511 (1.7)	9772 (1.6)	10398 (1.7)	9820 (1.8)	10014 (1.8)
	Stop	66337 (12.1)	69887 (12.3)	67003 (11.6)	69047 (12.7)	79559 (14)	82946 (14.4)	78366 (13.6)	77764 (14.3)	92096 (15)	89504 (14.8)	83014 (15.2)	89437 (16.1)
**Treatment incl. Thiazide**	Restart, complete	340753 (64.4)	338750 (63.3)	349567 (62.7)	317940 (60.9)	318632 (59.3)	320896 (58.4)	332020 (61.4)	307717 (62.0)	346769 (61.9)	331595 (61.7)	299645 (61)	295916 (59.9)
	Restart, intensified	17798 (3.4)	17323 (3.2)	18695 (3.4)	17210 (3.3)	17520 (3.3)	17266 (3.1)	18995 (3.5)	17836 (3.6)	17351 (3.1)	17165 (3.2)	16610 (3.4)	16040 (3.2)
	Restart, partial	88181 (16.7)	92875 (17.3)	101669 (18.2)	95795 (18.3)	102857 (19.1)	105630 (19.2)	93762 (17.3)	81703 (16.5)	93730 (16.7)	89079 (16.6)	79923 (16.3)	85568 (17.3)
	Switch, complete	7552 (1.4)	8135 (1.5)	9093 (1.6)	8971 (1.7)	8911 (1.7)	8735 (1.6)	8332 (1.5)	7403 (1.5)	7757 (1.4)	7977 (1.5)	7837 (1.6)	7442 (1.5)
	Switch, partial	9008 (1.7)	9462 (1.8)	11426 (2.0)	12336 (2.4)	11805 (2.2)	11514 (2.1)	11216 (2.1)	9872 (2.0)	9926 (1.8)	10579 (2.0)	10594 (2.2)	10313 (2.1)
	Stop	65842 (12.4)	68816 (12.9)	67297 (12.1)	70110 (13.4)	77976 (14.5)	85196 (15.5)	76395 (14.1)	71918 (14.5)	84802 (15.1)	80625 (15)	76442 (15.6)	78648 (15.9)

Annex 2 – Table 3. Distribution of treatment disruptions in patients using at least one antihypertensive drug, by sex or age

		**Mar 18' - May 18'**	**Jun 18' - Aug 18'**	**Sep 18' - Nov 18'**	**Dec 18' - Feb 19'**	**Mar 19' - May 19'**	**Jun 19' - Aug 19'**	**Sep 19' - Nov 19'**	**Dec 19' - Feb 20'**	**Mar 20' - May 20'**	**Jun 20' - Aug 20'**	**Sep 20' - Nov 20'**	**Dec 20' - Feb 21'**
**Men**	Restart, complete	578486 (61.5)	586812 (61.4)	601397 (61.2)	563585 (59)	575273 (59)	599621 (59.5)	609347 (60.6)	564792 (59.8)	622398 (60.3)	615383 (60.2)	558532 (59.2)	551193 (57.5)
	Restart, intensified	42443 (4.5)	42174 (4.4)	44666 (4.5)	42680 (4.5)	43287 (4.4)	42622 (4.2)	45288 (4.5)	43187 (4.6)	40417 (3.9)	42759 (4.2)	40807 (4.3)	38291 (4.0)
	Restart, partial	112823 (12.0)	117589 (12.3)	123112 (12.5)	119823 (12.5)	121638 (12.5)	126351 (12.5)	120297 (12.0)	111126 (11.8)	123289 (11.9)	119765 (11.7)	109600 (11.6)	119375 (12.4)
	Switch, complete	61515 (6.5)	58873 (6.2)	66967 (6.8)	73228 (7.7)	68280 (7.0)	64208 (6.4)	65341 (6.5)	63254 (6.7)	58431 (5.7)	62040 (6.1)	61579 (6.5)	62663 (6.5)
	Switch, partial	12400 (1.3)	12659 (1.3)	15012 (1.5)	16159 (1.7)	15071 (1.5)	14896 (1.5)	15077 (1.5)	14190 (1.5)	13887 (1.3)	15293 (1.5)	15287 (1.6)	15259 (1.6)
	Stop	133685 (14.2)	137075 (14.4)	131226 (13.4)	140363 (14.7)	151963 (15.6)	160469 (15.9)	149421 (14.9)	148214 (15.7)	174556 (16.9)	166326 (16.3)	157936 (16.7)	172554 (18.0)
**Women**	Restart, complete	730903 (62.5)	728127 (62.4)	743086 (61.9)	700852 (60.0)	715666 (59.0)	728523 (59.1)	748570 (61.0)	702335 (60.8)	790450 (61.5)	759904 (60.9)	689089 (59.7)	686499 (58.5)
	Restart, intensified	50071 (4.3)	47017 (4.0)	51119 (4.3)	48568 (4.2)	47949 (4.0)	47757 (3.9)	52866 (4.3)	49396 (4.3)	46832 (3.6)	48759 (3.9)	46295 (4.0)	44417 (3.8)
	Restart, partial	132752 (11.4)	137299 (11.8)	144891 (12.1)	139588 (11.9)	150464 (12.4)	152585 (12.4)	139784 (11.4)	127073 (11.0)	144855 (11.3)	138312 (11.1)	126860 (11.0)	141632 (12.1)
	Switch, complete	76449 (6.5)	72721 (6.2)	82397 (6.9)	88575 (7.6)	86652 (7.1)	80977 (6.6)	81838 (6.7)	77243 (6.7)	73482 (5.7)	78610 (6.3)	76806 (6.7)	76435 (6.5)
	Switch, partial	14678 (1.3)	14892 (1.3)	17160 (1.4)	18987 (1.6)	18257 (1.5)	17771 (1.4)	17930 (1.5)	16497 (1.4)	16527 (1.3)	17909 (1.4)	17687 (1.5)	17458 (1.5)
	Stop	164149 (14.0)	167388 (14.3)	162035 (13.5)	171675 (14.7)	193377 (16.0)	204591 (16.6)	187065 (15.2)	182165 (15.8)	213719 (16.6)	205085 (16.4)	196723 (17.1)	206080 (17.6)
**Under 65 years old**	Restart, complete	407469 (66.8)	410067 (66.6)	420294 (66.4)	392813 (64)	402076 (63.4)	413555 (63.6)	419847 (65.1)	391107 (64.2)	431417 (64.1)	423155 (64.5)	382070 (62.8)	384575 (62.2)
	Restart, intensified	23543 (3.9)	22723 (3.7)	24076 (3.8)	23462 (3.8)	24278 (3.8)	23717 (3.6)	25285 (3.9)	24522 (4.0)	23248 (3.5)	24218 (3.7)	23267 (3.8)	22338 (3.6)
	Restart, partial	46035 (7.5)	48268 (7.8)	51608 (8.1)	49335 (8.0)	50896 (8.0)	52064 (8.0)	49170 (7.6)	44792 (7.4)	50683 (7.5)	47718 (7.3)	44736 (7.4)	47058 (7.6)
	Switch, complete	33354 (5.5)	32098 (5.2)	36641 (5.8)	39537 (6.4)	38044 (6.0)	35329 (5.4)	35565 (5.5)	34577 (5.7)	33066 (4.9)	33971 (5.2)	33993 (5.6)	33999 (5.5)
	Switch, partial	4962 (0.8)	5247 (0.9)	6232 (1.0)	6965 (1.1)	6400 (1.0)	6174 (0.9)	6242 (1.0)	5726 (0.9)	5720 (0.9)	6234 (1.0)	6275 (1.0)	6155 (1.0)
	Stop	94529 (15.5)	97158 (15.8)	94458 (14.9)	101749 (16.6)	112790 (17.8)	119093 (18.3)	109178 (16.9)	108123 (17.8)	128562 (19.1)	120777 (18.4)	117847 (19.4)	124327 (20.1)
**At least 65 years old**	Restart, complete	899557 (60.0)	903248 (59.9)	921537 (59.5)	868557 (57.5)	884372 (56.9)	909820 (57.2)	932111 (58.7)	870566 (58.4)	975104 (59.2)	942434 (58.4)	858754 (57.7)	867004 (57.3)
	Restart, intensified	69238 (4.6)	66635 (4.4)	72035 (4.6)	68077 (4.5)	67285 (4.3)	67039 (4.2)	73493 (4.6)	68533 (4.6)	64628 (3.9)	68134 (4.2)	64458 (4.3)	61025 (4.0)
	Restart, partial	201560 (13.4)	208158 (13.8)	218690 (14.1)	212977 (14.1)	225625 (14.5)	231538 (14.6)	216441 (13.6)	198867 (13.3)	225373 (13.7)	220454 (13.7)	199731 (13.4)	216429 (14.3)
	Switch, complete	105138 (7.0)	99879 (6.6)	113331 (7.3)	123075 (8.1)	118008 (7.6)	110969 (7.0)	113115 (7.1)	107355 (7.2)	100341 (6.1)	109175 (6.8)	106472 (7.2)	107668 (7.1)
	Switch, partial	22343 (1.5)	22459 (1.5)	26218 (1.7)	28543 (1.9)	27450 (1.8)	27027 (1.7)	27458 (1.7)	25680 (1.7)	25605 (1.6)	28235 (1.7)	27790 (1.9)	27900 (1.8)
	Stop	202625 (13.5)	206686 (13.7)	197947 (12.8)	208995 (13.8)	230653 (14.8)	244047 (15.3)	224919 (14.2)	219624 (14.7)	255096 (15.5)	245642 (15.2)	231808 (15.6)	233379 (15.4)
